# Anemia, iron deficiency, and thalassemia among the Thai population inhabiting at the Thailand-Lao PDR-Cambodia triangle

**DOI:** 10.1038/s41598-022-22016-3

**Published:** 2022-11-04

**Authors:** Rossarin Karnpean, Nawinda Vanichakulthada, Wanwisa Suwannaloet, Ruttiya Thongrung, Sanita Singsanan, Nattapol Prakobkaew, Goonnapa Fucharoen, Supan Fucharoen

**Affiliations:** 1 Department of Pathology, Faculty of Medicine, Maha Chakri Sirindhorn Medical Center, Srinakharinwirot University, Nakhon Nayok, Thailand; 2 College of Medicine and Public Health, Ubon Ratchathani University, Ubon Ratchathani, Thailand; 3 Faculty of Allied Health Sciences, Burapha University, Bangsean, Chonburi Thailand; 4 Faculty of Associated Medical Sciences, Centre for Research and Development of Medical Diagnostic Laboratories, Khon Kaen University, Khon Kaen, 40002 Thailand

**Keywords:** Genetics, Diseases, Health care, Medical research, Risk factors

## Abstract

Anemia is a major public health problem in many areas of Southeast Asia. Ascertaining anemia and defining its underlying causes is essential for providing appropriate care, management, and establishment of a control program. Limited studies on these have been carried out on people living at the borders of Thailand, Lao PDR, and Cambodia. This cross-sectional study was done in four areas along the borders of Thailand, Lao PDR, and Cambodia. Blood specimens were collected from subjects aged 15–18 years in four districts including Kantharalak, Si Sa Ket province (n = 36), Nam Khun (n = 109), Nam Yuen (n = 98), and Na Chaluai (n = 128), Ubon Ratchathani province, Thailand. RBC parameters were recorded, and serum ferritin (SF) level was measured. Diagnosis of thalassemia and hemoglobinopathies was based on hemoglobin (Hb) and DNA analyses. Measurement of C-reactive protein was performed to exclude false-negative result of iron deficiency. The prevalence of anemia was found to be 25.1%. ID accounted for only 10.5%. Various types of thalassemia were identified in 67.7% of the subjects. The overall prevalence of thalassemia included 3.5% α^0^-thalassemia, 0.8% β-thalassemia, 47.7% Hb E, and 53.6% α^+^-thalassemia. The proportions of ID, thalassemia and combined ID and thalassemia among anemic subjects were 6.5%, 66.6%, and 20.4%, respectively. The results indicate that thalassemia and hemoglobinopathies rather than ID are major causes of anemia in Thailand-Lao PDR-Cambodia triangle. This information should prove useful for implementing an anemia control program in the regions.

## Introduction

Anemia is strictly defined as a decrease in red blood cell (RBC) mass. In anemia, a reduction in the number of RBCs transporting oxygen and carbon dioxide impairs the body's ability for gas exchange. The decrease may result from blood loss, increased destruction of RBCs (hemolysis), or decreased production of RBCs. Clinical symptoms of anemia vary, including mild pallor, weakness, shortness of breath, fatigue, and reduced work or productivity^1,2^. Anemia has multiple etiologies, including iron deficiency (ID), chronic inflammatory disorders, micronutrient deficiencies, parasitic infections, excessive bleeding, and congenital defects of Hb production^3^. Ascertaining anemia and defining its underlying causes is essential for providing appropriate care and management of the patients as well as the establishment of a control program.

The global prevalence of anemia is about 33% for all ages^4^. For that prevalence, about 50% of anemia came from ID. In 2019, the World Health Organization (WHO) reported the anemia prevalence in Thailand as 24% in women of reproductive age (15–49 years), 23.8% in non-pregnant women of reproductive age, 32.2% in pregnant women, 24.9% in children aged 6–59 months, and considered to be a moderate public health problem^5^. Thalassemia and hemoglobinopathies are a highly prevalent and significant contributors to anemia in Thailand^4,6^. It is noteworthy that the clinical severity of different genotypes of thalassemia varies widely, ranging from asymptomatic to severe form of anemia^7^. Therefore, it is essential to survey the thalassemia genotypes and their contributions to anemia in the Thai population. In 2017, a study in central Thailand revealed that iron deficiency anemia (IDA) is the major cause of anemia in educated young Thai women^8^. In contrast, several studies in the northeastern Thailand including the surveys in school children^9,10^, adolescent^11^, women of reproductive age^12^, pregnant women^6^, and elderly^13^ indicated similarly that thalassemia and hemoglobinopathies rather than ID were major causes of anemia. Recently, it has been found that 38.2% of community-dwelling elderly in Thailand had anemia and the presence of anemia was associated with an increased risk of mortality^14^. However, while many studies have been conducted in diverse geographical areas, limited studies have been conducted on thalassemia and ID in remote areas of Thailand, especially at the triangle of Thailand-Laos-Cambodia borders. In this report, we have carried out a cross-sectional survey on the prevalence of anemia, ID, and thalassemia among the Thai population living at the borders of Thailand, Lao PDR, and Cambodia. This wealth of information will serve as a more understanding of the region's real situation and help in development of an appropriate guideline for preventing and controlling anemia in the region.

## Materials and methods

### Subjects

**Figure 1:**
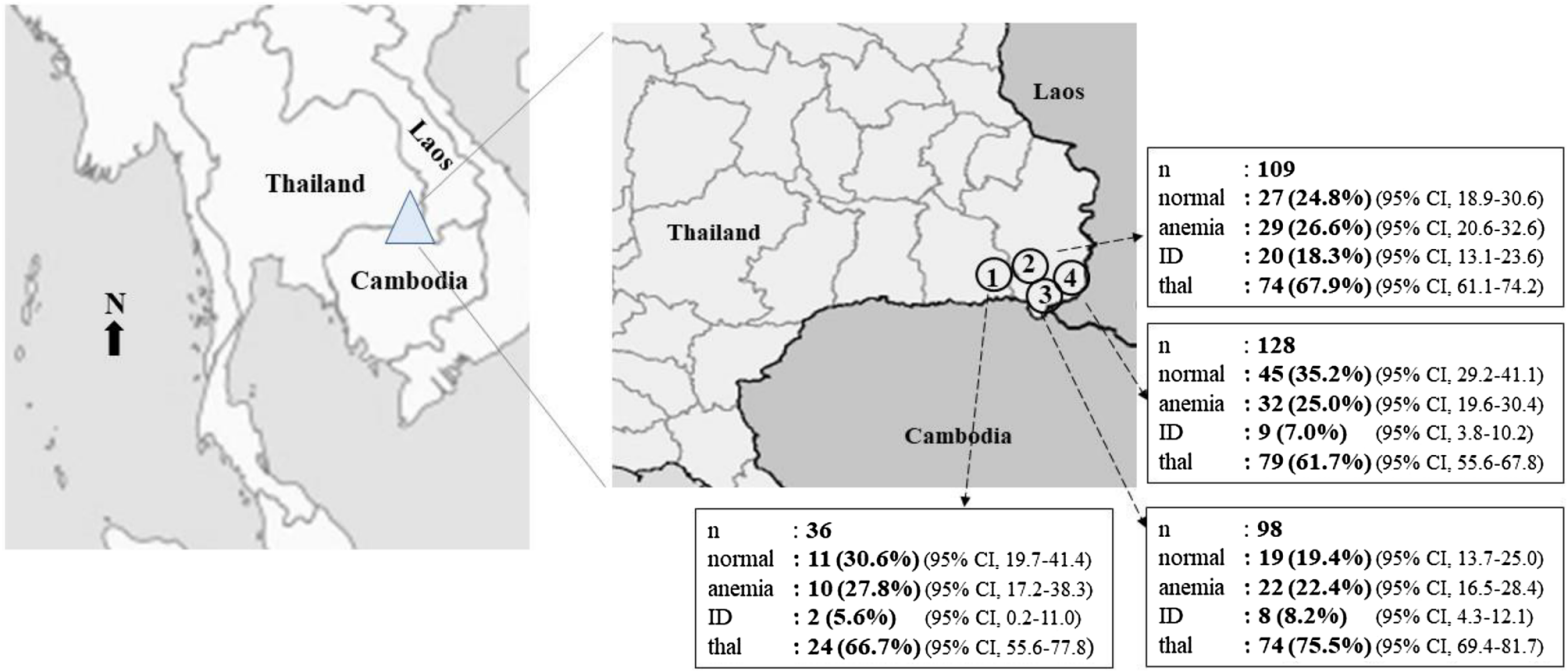
Map of Thailand, Lao PDR and Cambodia illustrating four districts at the Thailand-Lao PDR-Cambodia triangle where 371 specimens were recruited. ①, **②**, **③**, and **④** indicated Kantharalak, Nam Khun, Nam Yuen, and Na Chaluai districts, respectively. The prevalence of anemia, iron deficiency (ID), and thalassemia (thal) in each area were summarized in the corresponding box. Normal indicated subjects who had no anemia, ID and thalassemia.

This study was conducted in accordance with the Declaration of Helsinki and the study protocol was approved by the Institution Review Board (IRB) of Ubon Ratchathani University, Ubon Ratchathani, Thailand (UBU-REC-22/2559). The criteria for volunteer enrollment in this study were as follows; living in the districts of Thailand at the border of Thailand, Lao PDR, and Cambodia, being unrelated, and recognized as non-dual nationality. Based on the average prevalence of anemia among northeast Thai population of 21.1%^11^, and a total population of around 18,000 individuals of 15–18-year-old within the study areas, sample size estimation was calculated to be 252. Written informed consents were obtained from all subjects and the parents or guardians of the children under 18 years old. Exclusion criteria was subjects with inflammation or infection as judged by positive serum C-reactive protein (CRP). Seven subjects with positive serum CRP were excluded. Thus, a total of 371 subjects were included in the statistical analyses. The location of sampling areas and number of samples were illustrated in Fig. 1.

### Laboratory investigation

Complete blood count (CBC) was performed in routine practice at the College of Medicine and Public Health, Ubon Ratchathani University, by using the HmX Hematology Analyzer (Beckman Coulter, USA) within 24 h of blood collection. Hb analysis was done using capillary zone electrophoresis (Minicap: Sebia, Lisses, France). Serum ferritin (SF) was assayed using chemiluminescent immunoassay on the Syncron LXi^®^725 Access^®^ Clinical system (Beckman Coulter, USA). Based the WHO criteria, Hb levels of less than 13.0 g/dL in male and 12.0 g/dL in female were classified as anemia and SF level lower than 15.0 µg/L was identified as ID^15^. Measurement of CRP was performed by using CRP latex test kit (Plasmatec, UK). For globin gene genotyping, DNA was extracted from peripheral blood leukocytes using the GF1-blood DNA extraction kit (Vivantis Technologies Sdn Bhd, Selangor Darul Ehsan, Malaysia) and identifications of common α-thalassemia including α^0^-thalassemia (SEA & THAI deletions) and α^+^-thalassemia (− α^3.7^ & − α^4.2^) were routinely performed using the gap-PCR methods described elsewhere^16,17^. Hb Constant Spring and Hb Pakse′, the two α-hemoglobinopathies commonly found in the region were identified using a muliplex allele-specific PCR assay^18,19^. β-globin genotyping was performed using PCR based methods described elsewhere^20^.

### Data analysis

Data processing was done using IBM SPSS Statistic 20 software (SPSS Inc, Chicago, IL, USA). Descriptive statistics, i.e., percentage and the 95% confidence intervals, as well as mean and standard deviation, were used to describe the prevalence of ID, thalassemia as well as hematological values. To compare the difference in the proportions of thalassemia between the areas under study, the Z-test was applied. The Mann Whitney U test was used to compare the mean difference of hematological parameters between the two independent groups. Multiple logistic regression was applied to identify the risk factors associated with anemia, in which the independence variables included ID, different types of thalassemia, and combination of ID and thalassemia. A *P* value < 0.05 was considered statistical significance.

## Results

EDTA anticoagulated blood specimens were collected from 378 Thai subjects, aged 15–18 years who were living in the four areas: Kantharalak district of Si Sa Ket province, and Nam Khun, Nam Yuen, and Na Chaluai districts of Ubon Ratchathani province. Seven subjects with positive serum CRP were excluded. Thus, a total of 371 subjects were included in the statistical analysis (93 males and 278 females). The prevalence of anemia, ID and thalassemia observed in these subjects were shown in Fig. 1. Altogether, among 371 subjects aged 15–18 years, anemia was detected in 93 (25.1%) individuals. SF identified 39 cases (10.5%) with ID. Out of 371 subjects, 251 (67.7%) were found to carry thalassemia genes. A low prevalence of anemia and ID and a high prevalence of thalassemia were similarly observed in each studied area as shown in the figure.

Table 1 illustrated the 27 genotypes of thalassemia observed among 251 subjects with thalassemia. The three most common thalassemia genotypes found in Kantharalak, Nam Khun, Nam Yuen, and Na Chaluai were heterozygous α^+^-thalassemia (αα/− α^3.7^, β^A^/β^A^), double heterozygous for α^+^-thalassemia and Hb E (αα/− α^3.7^, β^A^/β^E^), and heterozygous Hb E (αα/αα, β^A^/β^E^). In addition, the genotypes of thalassemia disease were also observed, including one case each of the EABart’s disease or Hb H disease with Hb E heterozygote (–^SEA^/− α^3.7^, β^Α^/β^Ε^), Hb E-β^+^-thalassemia disease (αα/αα, β^−28^/β^Ε^), and Hb E-β^+^-thalassemia disease with heterozygous α^+^-thalassemia (αα/-α^3.7^, β^−28^/β^Ε^).

**Table 1:** Globin genotypes of 371 subjects inhabiting at the border region of Thailand, Lao PDR and Cambodia.

Thalassemia type	Genotype	Kantharalak		Nam Khun		Nam Yuen		Na Chaluai	
		n	%	n	%	n	%	n	%
Non thalassemia	1. αα/αα, β^A^/β^A^	12	33.3	35	32.1	24	24.5	49	38.3
Non-clinically significant thalassemia	2. αα/− α^3.7^, β^A^/β^A^	4	11.1	14	12.9	18	18.4	12	9.3
	3. αα/− α^4.2^, β^A^/β^A^	0	0	1	0.9	0	0	1	0.8
	4. αα/α^CS^α, β^A^/β^A^	2	5.6	6	5.5	6	6.1	6	4.7
	5. αα/α^PS^α, β^A^/β^A^	1	2.8	0	0	1	1.0	0	0
	6. α^3.7^/− α^3.7^, β^A^/β^A^	0	0	0	0	2	2.0	2	1.6
	7. α^CS^α/− α^3.7^, β^A^/β^A^	0	0	3	2.8	4	4.2	3	2.3
	8. α^PS^α/− α^3.7^, β^A^/β^A^	0	0	1	0.9	1	1.0	0	0
α^0^-thalassemia trait	9. αα/–^SEA^, β^A^/β^A^	2	5.6	3	2.8	1	1.0	2	1.6
Hb E trait	10. αα/αα, β ^A^/β^E^	3	8.3	18	16.5	11	11.2	21	16.4
Hb E trait with α^+^-thalassemia	11. αα/− α^3.7^, β^A^/β^E^	8	22.2	8	7.3	10	10.2	11	8.5
	12. αα/− α^4.2^, β^A^/β^E^	1	2.8	1	0.9	0	0	1	0.8
	13. αα/α^CS^α, β^A^/β^E^	2	5.6	7	6.4	2	2.0	6	4.7
	14. αα/α^PS^α, β^A^/β^E^	0	0	0	0	1	1.0	0	0
	15. α^3.7^/− α^3.7^, β^A^/β^E^	0	0	3	2.8	1	1.0	2	1.6
	16. α^CS^α/− α^3.7^, β^A^/β^E^	0	0	2	1.8	2	2.0	0	0
	17. α^CS^α/− α^PS^, β^A^/β^E^	0	0	0	0	0	0	1	0.8
Hb E trait with α^0^-thalassemia	18. αα/–^SEA^, β^A^/β^E^	0	0	0	0	1	1.0	3	2.3
Homozygous Hb E	19. αα/αα, β^E^/β^E^	1	2.8	4	3.7	4	4.2	4	3.1
Homozygous Hb E with α^+^-thalassemia	20. αα/− α^3.7^, β^E^/β^E^	0	0	1	0.9	3	3.1	2	1.6
	21. αα/α^CS^α, β^E^/β^E^	0	0	0	0	3	3.1	0	0
	22. α^CS^α/− α^3.7^, β^E^/β^E^	0	0	1	0.9	1	1.0	0	0
β-thalassemia trait	23. αα/αα, β^A^/β^E3.4 kb^	0	0	0	0	0	0	1	0.8
δβ-thalassemia trait	24. αα/αα, β^A^/δβ^0^	0	0	1	0.9	0	0	0	0
Thalassemia disease	25. –^SEA^/− α^3.7^, β^A^/β^E^	0	0	0	0	1	1.0	0	0
	26. αα/αα, β^− 28^/β^E^	0	0	0	0	1	1.0	0	0
	27. αα/− α^3.7^, β^− 28^/β^E^	0	0	0	0	0	0	1	0.8
**Total**		**36**	**100**	**109**	**100**	**98**	**100**	**128**	**100**

The prevalence and gene frequencies of non-clinically significant thalassemia and major thalassemia were described in Table 2. The overall prevalence of α^0^-thalassemia, β-thalassemia, Hb E, and α^+^-thalassemia (− α^3.7^, − α^4.2^,α^CS^α,·α^PS^α) were 3.5% (95%CI; 2.2–4.9), 0.8% (95%CI; 0.2–1.5), 47.7% (95%CI; 40.0–51.4), and 53.6% (95%CI; 50.0–57.3), respectively. The most common α^+^-thalassemia gene was − α^3.7^. Hb Constant Spring (Hb CS), Hb Pakse′ (Hb PS) and α^+^-thalassemia (4.2 kb deletion) were relatively rarer. The overall frequency of Hb Constant Spring, Hb Pakse′, and − α^4.2^ was similar in each area of the Thailand-Lao PDR-Cambodia triangle. A significant difference between gene frequency of − α^3.7^ in Nan Khun and Nam Yuen was observed (*P* value; 0.041). A significant difference was also observed in Nam Yuen and Na Chaluai (*P* value; 0.005). However, the prevalence and gene frequencies of major thalassemia were similar in the four districts. Hb E was the most common thalassemia found in Kantharalak, Nam Khun, Nam Yuen, and Na Chaluai, with a prevalence of 44.4%, 46.8%, 53.1%, and 45.3%, respectively. Fortunately, although Hb E is very common, very low prevalence of β-thalassemia was found. Interaction of Hb E and β-thalassemia would lead to the Hb E- β-thalassemia disease.

**Table 2:** Prevalence and gene frequency of thalassemia found among 371 subjects inhabiting at the border region of Thailand, Lao PDR and Cambodia (95% confidence interval in parentheses).

Type of Thalassemia	Kantharalak (n = 36, total 72 alleles)			Nam Khun (n = 109, total 218 alleles)			Nam Yuen (n = 98, total 196 alleles)			Na Chaluai (n = 128, total 256 alleles)		
	n	Prevalence	Gene frequency	n	Prevalence	Gene frequency	n	Prevalence	Gene frequency	n	Prevalence	Gene frequency
− α^3.7a^	12	33.3	0.167	36	33.3	0.165^c^	46	46.9	0.235^c, d^	37	28.9	0.145^d^
		(22.2–44.4)	(0.079–0.255)		(26.7–39.4)	(0.115–0.215)		(39.8–54.1)	(0.174–0.295)		(23.2–34.6)	(0.101–0.188)
− α^4.2^	1	2.8	0.014	2	1.8	0.009	0	0	0	2	1.1	0.008
		(− 1.1–6.7)	(− 0.014–0.041)		(0–3.7)	(− 0.004–0.222)					(0–3.1)	(− 0.003–0.019)
α^CS^	4	11.1	0.056	19	17.4	0.087	18	18.4	0.092	16	12.5	0.063
		(3.7–18.5)	(0.002–0.110)		(12.3–22.6)	(0.049–0.125)		(12.8–23.9)	(0.051–0.133)		(8.4–16.6)	(0.032–0.093)
α^PS^	1	2.8	0.014	1	0.9	0.005	3	3.1	0.015	1	0.8	0.004
		(− 1.1–6.7)	(− 0.014–0.041)		(− 0.4–2.2)	(− 0.005–0.014)		(0.6–5.5)	(− 0.002–0.033)		(− 0.3–1.9)	(− 0.004–0.012)
α^0^-thalassemia	2	5.6	0.028	3	2.8	0.014	3	3.1	0.015	5	4.7	0.023
		(0.2–11.0)	(− 0.011–0.067)		(0.5–5.0)	(− 0.002–0.030)		(0.6–5.5)	(− 0.002–0.033)		(2.0–7.3)	(0.005–0.042)
Hb E^a^	16	44.4	0.222	51	46.8	0.234	52	53.1	0.265	58	45.3	0.227
		(32.7–56.2)	(0.124–0.320)		(40.0–53.5)	(0.177–0.291)		(45.9–60.2)	(0.202–0.328)		(39.1–51.5)	(0.174–0.279)
β-thalassemia^b^	0	0	0	0	0	0	1	1	0.005	2	1.1	0.008
								(− 0.4–2.5)	(− 0.005–0.015)		(0–3.1)	(− 0.003–0.019)

The overall prevalence of α^+^-thalassemia (− α^3.7^, −  α^4.2^, α^CS^α, α^PS^α): 199/371 = 53.6% (95%CI: 50.0–57.3), α^0^-thalassemia: 13/371 = 3.5% (95%CI: 2.2–4.9), Hb E: 177/371 = 47.7% (95%CI: 40.0–51.4), and β-thalassemia: 3/371 = 0.8% 95%CI: (0.2–1.5).

^a^ Including individuals with heterozygous and homozygous states.

^b^ Including two individuals with − 28 (A > G) (HBB: c. − 78A > G) and one individual with 3.4 kb deletion (NC_000011.10: g.5224302-5227791del13490bp).

^c^ Significant difference between Nan Khun and Nam Yuen (*P* value; 0.041).

^d^ Significant difference between Nam Yuen and Na Chaluai (*P * value; 0.005).

Table 3 described the causes of anemia and hematological characteristics of 93 anemic subjects. The proportions of ID, thalassemia and combined ID & thalassemia among anemic subjects were 6.5%, 66.6% and 20.4%, respectively. The unknown cause of anemia was observed in six cases (6.5%). Comparison of hematological parameters in those four groups was examined in only female subjects due to the small number of male subjects. It is noted that subjects with thalassemia had significantly higher RBC count but lower MCV, MCH, and MCHC values, as compared to the ID group. Comparison of ID and combined ID & thalassemia, it was found that the latter had significantly higher RBC count and RDW value but lower Hb, MCV, MCH, and MCHC. The Hb and Hct parameters of combined ID & thalassemia were significantly lower than those with thalassemia alone, whereas the RDW was higher. The female subjects with thalassemia alone and combined ID & thalassemia had higher RBC count and lower MCV, MCH, and MCHC, when compared to those with anemia from other causes.

**Table 3:** Proportions and hematological characteristics of iron deficiency anemia (IDA), thalassemia and combined IDA and thalassemia among 93 anemic subjects inhabiting at the border region of Thailand, Lao PDR and Cambodia (hematological data expressed as mean ± SD and min–max in parentheses).

Anemic causes	n (%)	Sex (n)	RBC (10^12^/L)	Hb (g/dL)	Hct (%)	MCV (fL)	MCH (pg)	MCHC (g/dL)	RDW (%)
ID	6 (6.5)	Male (0)	–	–	–	–	–	–	–
		Female (6)	3.90 ± 0.19 ^a, b^ (3.77–4.13)	11.5 ± 0.4 ^b^ (11.2–11.9)	34.6 ± 1.3 (33.8–36.1)	89.0 ± 6.2^a, b^ (82.6–95.0)	29.6 ± 2.2 ^a, b^ (27.2–31.4)	33.2 ± 0.4 ^a, b^ (32.9–33.6)	13.4 ± 1.3 ^b^ (12.4–14.9)
Thalassemia	62 (66.6)	Male (8)	5.76 ± 0.96 (4.15–7.32)	12.0 ± 1.1 (9.7–12.9)	38.8 ± 3.1 (32.5–42.8)	68.6 ± 9.7 (50.3–78.6)	21.3 ± 3.2 (15.3–25.6)	30.9 ± 0.9 (29.9–32.5)	15.0 ± 2.3 (12.8–19.1)
		Female (54)	4.96 ± 0.52 ^a, d^ (4.15–6.40)	11.2 ± 0.7 ^c^ (7.9–11.9)	35.4 ± 2.7 ^c^ (26.9–46.7)	71.9 ± 6.8 ^a, d^ (56.2–83.6)	22.9 ± 2.6 ^a, d^ (16.4–27.4)	31.8 ± 1.3 ^a, d^ (25.3–33.7)	14.5 ± 2.5 ^c^ (11.5–24.5)
ID and thalassemia	19 (20.4)	Male (1)	4.55	9.6	30.8	67.2	21.0	31.0	17.0
		Female (18)	4.86 ± 0.52 ^b, e^ (3.96–6.06)	10.7 ± 0.9 ^b, c, e^ (8.8–11.9)	33.6 ± 2.5 ^c^ (28.6–38.7)	69.7 ± 7.3 ^b, e^ (55.2–81.6)	22.2 ± 2.8 ^b, e^ (16.9–27.0)	31.8 ± 0.9 ^b, e^ (29.6–33.5)	16.2 ± 2.5 ^b, c, e^ (12.9–22.1)
Other	6 (6.5)	Male (0)	–	–	-–	–	–	–	–
		Female (6)	3.99 ± 0.13 ^d, e^ (3.80–4.18)	11.6 ± 0.3 ^e^ (11.2–11.9)	34.8 ± 0.7 (34.1–36.1)	87.2 ± 4.1 ^d, e^ (82.7–95.0)	29.1 ± 1.3 ^d, e^ (27.2–31.4)	33.3 ± 0.4 ^d, e^ (32.9–34.1)	12.9 ± 1.0 ^e^ (11.9–14.9)
Total	93 (100)	Male (9)	5.63 ± 0.93 (4.15–7.32)	11.8 ± 1.2 (9.6–12.9)	38.0 ± 3.7 (30.8–42.8)	68.6 ± 8.5 (50.3–78.6)	21.3 ± 2.9 (15.3–25.6)	31.0 ± 0.8 (29.9–32.5)	15.2 ± 2.2 (12.8–19.1)
		Female (84)	4.85 ± 0.60 (3.77–6.42)	11.1 ± 0.8 (7.9–11.9)	34.9 ± 2.6 (26.9–46.7)	72.8 ± 8.4 (55.2–95.0)	23.3 ± 3.2 (16.4–31.4)	32.0 ± 1.2 (25.3–34.1)	14.8 ± 2.5 (11.5–24.5)

^a^ Significant difference between iron deficiency and thalassemia female subjects (*P* < 0.05).

^b^ Significant difference between iron deficiency and combined iron deficiency/thalassemia female subjects (*P* < 0.05).

^c^ Significant difference between thalassemia and combined iron deficiency/thalassemia female subjects (*P* < 0.05).

^d^ Significant difference between thalassemia and other anemic female subjects (*P* < 0.05).

^e^ Significant difference between combined iron deficiency/thalassemia and other anemic female subjects (*P* < 0.05).

Table 4 illustrated contributing factors associated with anemia examined in our study using multiple logistic regression analysis. The influence of ID, different types of thalassemia, and combination of ID and thalassemia was evaluated on 353 subjects. Of these, 18 participants could not be grouped into appropriate thalassemia type were excluded from multiple logistic regression analysis due to small sample sizes. As shown in the table, all factors evaluated were statistically significant. Based on odds ratios (OR), the participants with ID, two α-gene defects, and homozygous Hb E were respectively 17.0, 34.0, and 13.9 times more likely to suffer from anemia than those without ID and thalassemia. The greatest significant association with anemia was observed in subjects with combined ID & thalassemia with OR of 40.4 (95%CI: 11.1–154.5).

**Table 4:** Logistic regression analysis for anemia among subjects inhabiting at the border region of Thailand, Lao PDR and Cambodia.

Variable	n	Non-anemia	Anemia	Odds ratio	95% CI	*P*-value
Non-ID, Non-thalassemia (reference)	108	102	6			
**ID**	12	6	6	17.0	3.3–84.9	< 0.001
**Thalassemia type**						
One α-gene defect^a^	65	50	15	5.1	1.7–16.9	< 0.001
Two α-gene defects^b^	21	7	14	34.0	8.6–139.3	< 0.001
Heterozygous Hb E either with or without α-gene defect	100	78	22	4.8	1.8–15.1	< 0.001
Homozygous Hb E either with or without α-gene defect	20	11	9	13.9	3.5–55.7	< 0.001
**Combined ID and thalassemia**^**c**^	27	8	19	40.4	11.1–154.5	< 0.001

^a^ Including heterozygous α^+^-thalassemia (− α^3.7^), heterozygous α^+^-thalassemia (− α^4.2^), heterozygous Hb CS, heterozygous Hb PS.

^b^ Including homozygous α^+^-thalassemia (− α^3.7^), compound heterozygous α^+^-thalassemia (− α^3.7^)/Hb CS, compound heterozygous α^+^-thalassemia (− α^3.7^)/Hb PS, heterozygous α^0^-thalassemia (–^SEA^).

^c^ Including heterozygous α^+^-thalassemia (− α^3.7^), heterozygous α^+^-thalassemia (− α^4.2^), heterozygous Hb CS, compound heterozygous α^+^-thalassemia (− α^3.7^)/Hb CS, heterozygous α^0^-thalassemia (–^SEA^), heterozygous Hb E either with or without α-gene defect, homozygous Hb E either with or without α-gene defect.

## Discussion

To the best of our knowledge, this is the first cross-sectional survey on the prevalence of anemia, ID, and thalassemia in the Thai population inhabiting at the border of Thailand, Lao PDR, and Cambodia, rural districts in the lower part of northeast Thailand. Subjects were adolescents aged 15–18 years who should be aware of health problems associated with anemia and poor academic performances in areas such as cognitive function, mathematics score, memory test, attention and verbal learning, and intelligent quotient test^21,22^. This study revealed that the overall prevalence of anemia in the studied subjects was 25.1%, quite similar to those of the previous studies in adolescents at two other provinces in northeast Thailand^11^, in educated young Thai women^8^, and in women of reproductive age^12^. Data also demonstrated that the prevalence of anemia in each of the four studied areas was similar (Fig. 1). However, it is unexpected that in these remote areas of Thailand-Lao PDR-Cambodia triangle, only 39 out of 371 participants (10.5%) had ID. This represented a lower prevalence as compared to the previous studies in educated young Thai women^8^, adolescents in northeast Thailand^11^, and women of reproductive age^12^. In contrast, this ID prevalence was higher than in a study on school children from northeast Thailand^9^, especially in school children from poor rural subdistricts in Ubon Ratchathani, the same province of this study^11^. Interestingly, among 93 anemic subjects, IDA was found to be only 6.5%, whereas thalassemia was identified at the highest prevalence (66.6%). Of noted, 20.4% of anemic subjects had combined ID and thalassemia (Table 3). Clearly, these results confirmed that ID was not a major cause of anemia in the population inhabiting at the Thailand-Lao PDR-Cambodia triangle. Besides, 40 participants in our study even had iron overload (SF concentration > 150 μg/L), and 28 of them had thalassemia (data not shown). However, ID is still an essential factor associated with anemia in the region. As shown in Table 4, by multiple logistic regression analysis, ID was the potential risk factor of anemia (OR = 17.0, 95%CI = 3.3–84.9). In addition, concomitant of ID and thalassemia served more severe anemia than ID or thalassemia alone (Table 3) and exhibited the greatest association with anemia (OR: 40.4, 95%CI: 11.1–154.5) (Table 4).

Our study draws attention to the fact that thalassemia and hemoglobinopathies are highly prevalent and heterogeneous in the area under study in that 67.7% (251/371) of the participants were found to carry thalassemia genes with as many as 27 different genotypes (Table 1). The overall prevalence of α^+^-thalassemia, α^0^-thalassemia, Hb E, and β-thalassemia were similar to those described in the previous studies in northeast Thailand, Lao PDR, and Cambodia ^23–25^. The high prevalence of α^+^-thalassemia and Hb E and a low prevalence of β-thalassemia were similarly observed in the four studied areas (Table 2). This confirmed that thalassemia and hemoglobinopathies, especially Hb E and α-globin gene defects are major public health problems in this area of Thailand-Lao PDR-Cambodia triangle. In fact, this area has been doubleted the Hb E triangle where the prevalence of Hb E in the population could be as high as 50%^26,27^. From this cross-sectional survey, we even found three cases of thalassemia disease, including one participant with the AEBart’s disease (genotype: –^SEA^/− α^3.7^, β^Α^/β^Ε^), and two patients with β-thalassemia/Hb E disease [genotypes: (αα/αα, β^−28^/β^Ε^) and (αα/− α^3.7^, β^−28^/β^Ε^)]. Following up on these three patients revealed that although with these thalassemic diseases they all had mild non-transfusion dependent thalassemia phenotype without serious health problems^28,29^.

Of interest is the results of multiple logistic regression analysis which indicated that either one α-gene defect, two α-gene defects, Hb E trait, or homozygous Hb E were important risk factors of anemia in this studied population. The findings of greater association with anemia of two α-globin gene defects (OR: 34.0, 95%CI: 8.6–139.3) and homozygous Hb E (OR: 13.9, 95%CI: 3.5–55.7) (Table 4) agreed with the findings in previous studies conducted among pregnant women, and women of reproductive age in northeast Thailand^6,12^. In addition, previous study in pregnant women has also demonstrated that iron supplementation during pregnancy is not beneficial for pregnant women who are homozygous for Hb E but a routine intervention should not cause iron overload as judged from short observation period^30^. This is very important information for establishment of anemia control program. As for Thailand^23,26^, thalassemia and hemoglobinopathies are also common in Cambodia and Lao PDR^31–34^. All these findings underline the importance of identification of α-globin gene defect and Hb E and the risk of developing anemia in these countries. Although other causes of anemia, not examined in this study, including deficiency of vitamin B12 or folate may modify our data, these are unlikely as most of these micronutrient deficiencies are rarely found in Thailand. However, possible limitation of this study may be related to the regression model which examined crude OR because we could not adjust some potential confounders such as age, sex, body mass index (BMI)^14^, history of recent blood donation, the length of menstruation, and amount of blood loss per day during menstruation.

Nonetheless, our study indicated that thalassemia and hemoglobinopathies rather than ID are major causes of anemia in Thai people living at the Thailand-Lao PDR-Cambodia triangle. However, ID is still important in this region where thalassemia and hemoglobinopathies are highly prevalent, as combined ID and thalassemia, representing 20.4% in this study, exhibited apparently more severe anemia as compared to ID or thalassemia alone (Table 3). This information is essential for planning of anemia control in the region. It is conceivable that thalassemia and hemoglobinopathies should be taken into consideration in the anemia control program. Therefore, in addition to improvement of socioeconomic status and health education, population screening of thalassemia and hemoglobinopathies^35,36^ should be included in the public health strategies for control of anemia in the region.

## Data Availability

All data generated or analyzed during this study are included in this published article.
